# Differences in Hip Muscle Strength and Static Balance in Patients with Transfemoral Amputations Classified at Different K-Levels: A Preliminary Cross-Sectional Study

**DOI:** 10.33137/cpoj.v5i1.37456

**Published:** 2022-01-12

**Authors:** S John, K Orlowski, K.U. Mrkor, J Edelmann-Nusser, K Witte

**Affiliations:** 1 Department of Sports Science, Faculty of Humanities, Otto von Guericke University, Magdeburg, Germany.; 2 Department of Computer Science and Media, Brandenburg University of Applied Sciences, Brandenburg an der Havel, Germany.

**Keywords:** Amputation, K-level, Balance, Transfemoral Amputation, Muscle Strength, Residual Limb, Static Balance

## Abstract

**BACKGROUND:**

Following amputation, patients with lower limb amputations (LLA) are classified into different functional mobility levels (K-levels) ranging from K0 (lowest) to K4 (highest). However, K-level classification is often based on subjective criteria. Objective measures that are able to differentiate between K-levels can help to enhance the objectivity of K-level classification.

**OBJECTIVE(S):**

The goal of this preliminary cross-sectional study was to investigate whether differences in hip muscle strength and balance parameters exist among patients with transfemoral amputations (TFA) assigned to different K-levels.

**METHODOLOGY:**

Twenty-two participants with unilateral TFA were recruited for this study, with four participants assigned to K1 or K2, six assigned to K3 and twelve assigned to K4. Maximum isometric hip strength of the residual limb was assessed in hip flexion, abduction, extension, and adduction using a custom-made diagnostic device. Static balance was investigated in the bipedal stance on a force plate in eyes open (EO) and eyes closed (EC) conditions. Kruskal-Wallis tests were used to evaluate differences between K-level groups.

**FINDINGS:**

Statistical analyses revealed no significant differences in the parameters between the three K-level groups (p>0.05). Descriptive analysis showed that all hip strength parameters differed among K-level groups showing an increase in maximum hip torque from K1/2-classified participants to those classified as K4. Group differences were also present in all balance parameters. Increased sway was observed in the K1/2 group compared to the K4 group, especially for the EC condition.

**CONCLUSION:**

Although not statistically significant, the magnitude of the differences indicates a distinction between K-level groups. These results suggest that residual limb strength and balance parameters may have the potential to be used as objective measures to assist K-level assignment for patients with TFA. This potential needs to be confirmed in future studies with a larger number of participants.

## INTRODUCTION

Amputations of the lower limbs, especially transfemoral (TF) or transtibial (TT) amputations have a severe impact on the patient's life. The irreversible loss of an extremity affects physical integrity and leads to social and psychological burdens.^1^ Following amputation, rehabilitation programs and proper prosthetic fitting, as well as prosthetic usage, are important factors for improving the quality of life of the patients.^2^ The use of a prosthesis has been associated with higher physical function, gain in independence and increased self-esteem.^3^

The prescription of the type of prosthesis and its specified components as well as the financial coverage by health insurance are based on the expected functional mobility of the patient.^4^ To identify functional mobility, several classification systems exist to assign patients with lower limb amputations (LLA) to different mobility levels.^4,5^ In the United States, the Medicare's Functional Classification Level (MFCL) distinguishes patients with LLA into five functional levels ranging from K0 (lowest) to K4 (highest). This classification, which is intended to reflect the individual's abilities to ambulate with the prosthesis, strongly influences the selection and assignment of the different prosthetic components. Patients with LLA classified as K2 will not have the possibility to receive high functioning prosthesis components as patients classified at K4. In Germany, a similar classification system is used with the same categories as the MFCL system. The assignment into the different levels is based on the so-called profile survey sheet, in which doctors or orthopedic technicians subjectively evaluate abilities concerning functional mobility.^6^ As objective parameters, only the range of motion (ROM) of joints of the lower extremities are documented. Further objective evaluation criteria are missing. Due to the relevance of K-level assignment for the patients with LLA, the lack of objectivity has been recognized and 75% of orthopedic technicians would support additional objective measures to improve the subjectivity of K-level classification.^7^ In a recent study, Sions et al. emphasized the necessity of reliable and valid objective measures to differentiate between K-level classifications.^8^

Addressing the subjectivity of the existing K-level classification, Gailey et al. were the first to develop a clinical tool, the Amputee Mobility Predictor (AMP),^4^ to objectively assess the patient's functional abilities. The AMP consists of 21 ambulation and balance tasks with and without prostheses, which are individually rated by an examiner using a point system. The AMP was shown to have the potential to distinguish between K-levels.^4,9^ In two recent papers, physical performance tests were performed and tested if they are suitable measures to improve the objectivity of K-level assignment.^8,10^ Differences between patients with LLA classified as K3 and K4 were seen in the Timed Up and Go test and the 6-minute walk test.^8^ In the study of Beisheim et al., functional strength and dynamic balance tests were performed with patients with LLA. K4-classified patients showed higher functional strength and better dynamic balance when compared to participants classified as K3.^10^ These studies show that walking tests, as well as functional tests, may help to assign patients with LLA to the different K-levels.

Performance in functional tests and walking tests are often associated with lower limb strength. Several studies have demonstrated that patients with transfemoral amputation (TFA) have significantly reduced strength in the residual limb compared to the sound leg as well as to controls.^11,12^ The muscles surrounding the hip are important to stabilize the pelvis during standing and locomotion. Weak hip abductors are one cause of the compensatory trunk shifting over the prosthetic side^13^ and poor balance performance.^14^ In a recent review, Hewson et al. concluded that muscle strength deficits exist in lower limb prosthesis users and contribute to balance and mobility impairments.^12^ In patients with TFA, these strength deficits are particularly pronounced in the hip of the residual limb.^12^

However, no study has included standardized hip strength tests of the residual limb as possible measures for assisting to objectify K-level classification. Objective evaluation methods must be tested for their suitability in assisting in K-level assignment, particularly methods that evaluate lower limb strength and balance. Therefore, the goal of this preliminary study was to investigate whether there are differences in hip muscle strength of the residual limb as well as differences in static balance parameters among patients with TFA assigned to different K-levels. The authors hypothesized that participants classified at higher K-levels would demonstrate higher performance on the strength and balance tests than participants that were assigned to lower K-levels.

## METHODOLOGY

### Participants

Participants were recruited from January 2018 to September 2019 through calls and articles in official journals of amputee organizations as well as in a local newspaper. Inclusion criteria were a unilateral transfemoral amputation with a post-amputation time of at least one year, an age ≥18 years and the current use of the prosthesis. Due to the measurement setup, one further inclusion criterion was a minimum residual limb length of 15 cm. Participants were excluded if the amputation was caused by diabetes mellitus, or if they had open wounds, edema, or acute pain in the residual limb. All participants gave written consent to participate in this study after being informed about the procedure and its purpose. The study was approved by the local ethics committee of the Otto von Guericke University Magdeburg and carried out in line with the Declaration of Helsinki (no. of vote: 31/18 on March 19, 2018).

### Measurement protocol

For this cross-sectional study, the participants attended a single testing session, in which all measurements were conducted. Measurements were performed in two institutions, University of Magdeburg and University of Applied Sciences Brandenburg, which were equipped with the same measurement systems. Prior to physical performance tests, demographic and anthropometric data were collected and participants were asked to answer amputation related questions (e.g. type of prosthesis, years of using the prosthesis, and K-level assignment). The K-level assignment was obtained from medical records in collaboration with the respective orthopedic technician. The physical performance tests included isometric strength tests of the hip muscles of the residual limb as well as examinations of static balance. The strength assessment of the hip muscles was performed without the prosthesis whereas the static balance tests were performed with the prosthesis.

### Maximum isometric hip strength analysis

**Figure 1:**
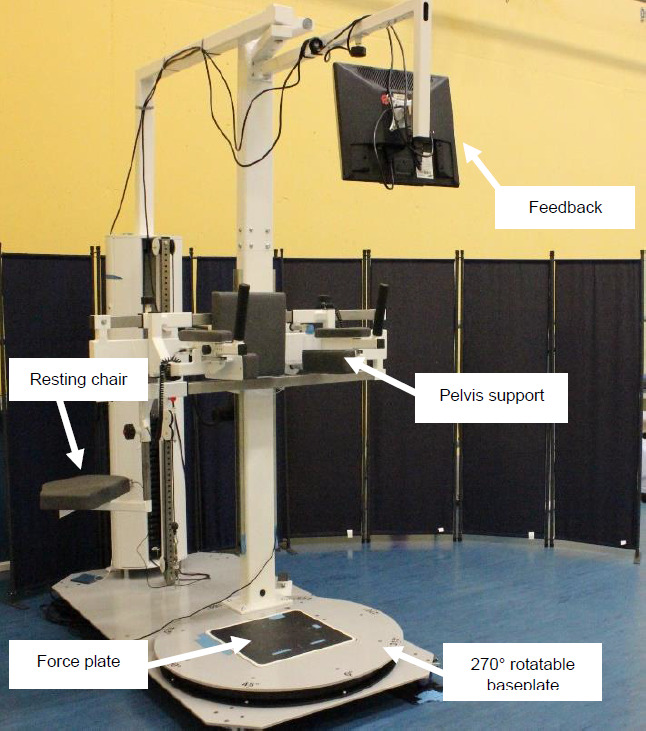
The sensor-based diagnostic device in the overall display.

The measurement of the maximum isometric strength of the hip muscles of the residual limb was performed in a custom-made diagnostic device (**Figure 1**). This diagnostic device was built specifically for patients with LLA. An individually adjustable pelvic support provides stability and safety during the measurements. The 270° rotatable base plate enables hip muscle strength diagnostics in different directions (hip flexion, extension, abduction and adduction) while participants do not need to change position within the device. An additional resting chair, which can be slid under the participants, is integrated into the device to provide relief of the standing leg between examinations.

**Figure 2:**
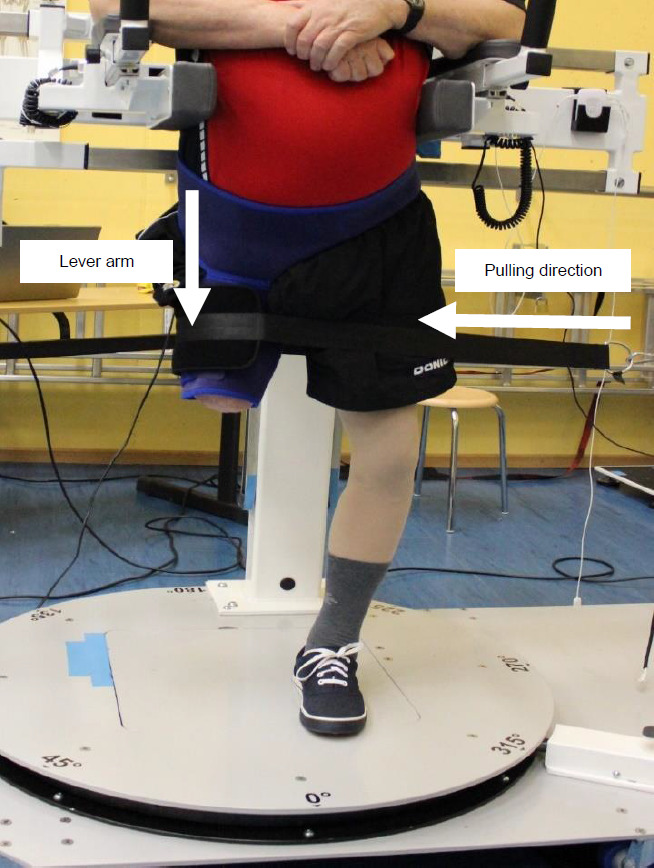
Setup for measuring strength in hip abduction in the neutral hip position.

For the strength measurement, participants were standing in an upright position supported by the pelvis support without the prosthesis. A neoprene brace was placed around the residual limb (**Figure 2**). This brace served as an attachment possibility for the cuff of the hauling rope. A force transducer (Hottinger Baldwin Messtechnik GmbH, Darmstadt, Germany) integrated into the hauling rope was used to measure the isometric strength at a sampling rate of 1000 Hz for hip flexion, extension, abduction, and adduction in the neutral hip position (vertical position of the thigh perpendicular to the pelvis). Before the measurements, participants were asked to familiarize themselves with the setup. For each motion direction, one submaximal test (pretest) and three maximum tests (main tests) were performed with one minute of rest between trials. Participants were instructed to successively build up strength and pull maximally without an abrupt push. They could follow their current measured strength values live on the screen during the measurement. The maximum achieved strength value from the pretest was visualized on the screen as a threshold value and participants were verbally encouraged to exceed this in the main tests. The threshold value was readjusted after exceeding the previous threshold value to ensure that the maximum possible force value was reached within the three main tests. The distance between the greater trochanter and the point of applied force (center of the cuff) served approximately as the lever arm (**Figure 2**).

Data were further processed in Matlab (Version 2018b, The MathWorks Inc., Natick, MA) and filtered with a 4th order Butterworth low-pass filter (5 Hz). Torques for each motion direction were calculated from the force and the lever arm and normalized to the body mass of the participants. Out of the three main trials for each motion direction, the trial with the highest torque was used for further analyses.

The reliability of the isometric hip strength measurement of the diagnostic device had been investigated in a test-retest design before the examinations. The calculated intraclass correlation coefficients (ICCs) showed values ranging from 0.85 to 0.95 for the isometric hip strength measurement (hip flexion, extension, abduction, and adduction). According to Koo and Li, these ICCs indicate good to excellent reliability.^15^ These results suggest that the custom-made diagnostic device provides an environment to reliably quantify maximum isometric hip strength.

### Balance assessment

Static balance was assessed in two different conditions: bipedal stance with eyes open (EO) and bipedal stance with eyes closed (EC). Before generating balance data, the prosthetic socket comfort was determined with the Prosthetic Socket Fit Comfort Score^16^ as poor socket fit might influence static balance parameters. Socket score has been deemed a valid and reliable outcome measurement.^16^ The participants were asked to rate the comfort of their socket on a scale from 0 to 10 with 0 being the most uncomfortable and 10 being the most comfortable socket imaginable. Mean comfort scores were between 7 and 8 points for all K-level groups.

For the bipedal stance, subjects were instructed to stand hip-width apart on a 45x45 cm force plate (PLUX-Wireless Biosignals S.A, Lisbon, Portugal) with the arms hanging down at the sides and to remain as still as possible. For the EO conditions, participants were asked to focus on a fixed point at eye-level on the wall in front of them whereas for the EC condition the participants closed their eyes. Prior to collecting data, participants practiced both poses for a few seconds. For safety reasons, an examiner stood near the participants during the entire familiarization and measurement period. Once the familiarization period was over, two trials with a duration of twenty seconds were recorded for both conditions with a sampling frequency of 250 Hz. Balance data were further processed using Matlab and filtered applying a 4th order Butterworth low-pass filter with a 10 Hz cut-off frequency. The total length of the center of pressure (COP) during the two standing conditions was computed as well as the maximum and mean deviations in mediolateral (ML) and anteroposterior (AP) directions. These COP-based measures have been used in most studies examining static balance in participants with LLA.^17^

### Data analysis

Statistical analyses were performed using IBM SPSS Statistics 26 (IBM SPSS, Armonk, NY). Based on the K-level assignment, participants were divided into groups. For each K-level group, descriptive statistics were determined for all anthropometric, demographic and measurement variables. Due to the small sample size of participants and the unequal distribution of participants across K-levels, variables were described using the median and interquartile range (IQR: 25th percentile, 75th percentile). To detect differences between K-level groups, Kruskal-Wallis tests were performed for each variable. Pairwise posthoc comparisons with Bonferroni correction for multiple testing followed where appropriate. The significance level was set at p< 0.05.

## RESULTS

### Participants

Twenty-two participants fulfilled the inclusion criteria and were considered for the study. All participants were able to complete all tests and were included in the data analysis. In **Table 1**, participants' anthropometric and demographic data are represented according to the K-level assignment. Due to the small numbers of patients classified as K1 or K2, they were combined as one group. The Kruskal-Wallis test showed that age differed significantly between K-level groups. Posthoc tests revealed that participants of the K4 group were significantly younger than the ones of the K1/2 group (p=0.03). The K4 group was not only younger on average, but also had a longer residual limb length and the amputation had not occurred as long ago as for K1/2 and K3-classified participants.

**Table 1:** Anthropometric and demographic data of the participants presented as median and IQR (25^th^ percentile, 75^th^ percentile).

	K1/2 (n=4)	K3 (n=6)	K4 (n=12)	p-value (Kruskal-Wallis)
**Age [yrs.]**	75.0^a^ (53.8, 80.5)	61.0 (51.0, 76.8)	51.5^a^ (36.3, 60.8)	0.04*
**Sex (m=male, f=female)**	4 m, 0 f	4 m, 2 f	12 m, 0 f	–
**BMI [kg/m2]**	28.8 (23.9, 30.1)	25.8 (23.8, 26.7)	26.6 (24.8, 30.5)	0.40
**Residual limb length [m]**	0.22 (0.22, 0.39)	0.32 (0.26, 0.35)	0.38 (0.28, 0.43)	0.18
**Years since amputation [yrs.]**	28.5 (3.0, 67.5)	19.0 (7.3, 35.5)	8.5 (4.3, 24.3)	0.63

^*^Significant across groups (*p*<0.05)

^a^Significant difference between the K1/2 and the K4 group

### Isometric hip strength of the residual limb

**Figure 3:**
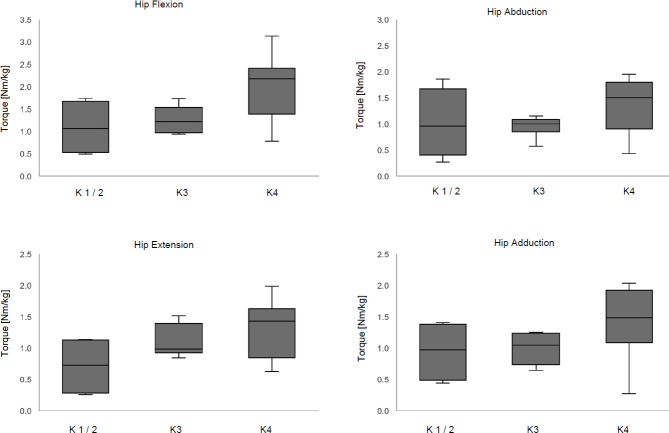
Maximum hip torque for the four movement directions of the three K-level groups.

The results of the hip muscle strength test of the residual limb are shown in **Table 2**. A significant difference across K-level groups was only detected for hip flexion (p=0.04). The posthoc tests did not reveal significant differences between the individual groups (p>0.05). If the medians of the parameters are considered, an increase in maximum hip torque can be observed from participants classified as K1/2 to those classified as K4. Differences were especially visible between the K3 and K4 groups with a mean difference of 0.97 Nm/kg for hip flexion, 0.51 Nm/kg for hip abduction, 0.45 Nm/kg for hip extension and 0.44 Nm/kg for hip adduction. A graphical representation of the data in form of boxplots is shown in **Figure 3**.

**Table 2:** Maximum hip torque presented as median and IQR (25^th^ percentile, 75^th^ percentile).

Hip Torque	K1/2	K3	K4	p-value (Kruskal-Wallis)
Hip flexion [Nm/kg]	1.06 (0.53, 1.68)	1.21 (0.97, 1.53)	2.18 (1.39, 2.42)	0.04^*^
Hip abduction [Nm/kg]	0.96 (0.41, 1.67)	1.00 (0.85, 1.09)	1.51 (0.91, 1.80)	0.32
Hip extension [Nm/kg]	0.73 (0.28, 1.13)	0.98 (0.93, 1.39)	1.43 (0.85, 1.63)	0.09
Hip adduction [Nm/kg]	0.98 (0.49, 1.38)	1.05 (0.74, 1.24)	1.49 (1.09, 1.92)	0.07

^*^Significant across groups (p<0.05)

### Static balance

**Figure 4:**
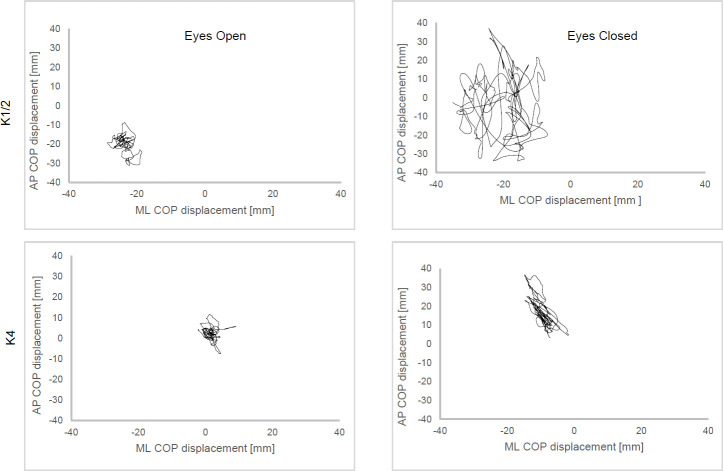
Examples of COP sway of one K1/2-participant compared to one classified as K4 in the eyes open and eyes closed condition.

The parameters from the examination of the static balance are presented in **Table 3**. For neither the EO nor the EC condition, the Kruskal-Wallis tests revealed significant differences in the parameters across the three K-level groups (p>0.05). The descriptive analysis showed that differences in the medians were especially seen between K1/2 classified participants and the ones assigned to K4. For the EO condition, the mean and maximum deviation in ML and AP directions decreased from K1/2 to the K4 group. For the maximum deviation in ML and AP directions, a mean difference of 8 mm and 6 mm was determined between K1/2 and K4. For the EC condition, differences between groups became more evident. For the COP length, the mean difference between the K1/2 group and K4 group was 340 mm, and the mean differences of the maximum excursions in ML and AP directions were 23 mm and 20 mm, respectively. **Figure 4** shows an example of sway paths from one participant of the K1/2 group and one K4-classified participant in both EO and EC conditions. An increase in sway from the EO condition to the EC condition is visible for both K-groups, although the increase is considerably more pronounced for the K1/2-participant than for that of the participant of the K4 group.

**Table 3:** COP parameters of the bipedal stance presented as median and IQR (25^th^ percentile, 75^th^ percentile).

Eyes open (EO)	K1/2	K3	K4	p-value (Kruskal-Wallis)
**COP length [mm]**	292.7 (243.3, 684.4)	244.3 (192.7, 456.5)	270.5 (217.1, 343.2)	0.54
Mean Dev. ML [mm]	4.2 (2.2, 5.9)	3.2 (2.7, 4.8)	3.2 (1.8, 4.2)	0.79
Mean Dev. AP [mm]	4.7 (3.5. 6.6)	4.0 (3.5, 4.7)	3.4 (2.4, 5.3)	0.50
Max. Dev. ML [mm]	21.0 (11.8, 29.3)	14.7 (11.8, 19.0)	12.8 (10.4, 20.1)	0.59
Max. Dev. AP [mm]	24.7 (19.7, 29.7)	17.8 (16.2, 23.9)	18.8 (12.8, 23.0)	0.23
**Eyes closed (EC)**				
COP length [mm]	925.0 (488.9, 1386.7)	624.5 (442.5, 993.8)	583.9 (387.7, 873.4)	0.50
Mean Dev. ML [mm]	8.0 (5.3, 10.2)	3.6 (2.9, 5.3)	3.4 (2.3, 7.6)	0.19
Mean Dev. AP [mm]	12.0 (6.5, 16.6)	9.1 (5.5, 9.9)	8.1 (5.7, 9.5)	0.41
Max. Dev. ML [mm]	38.0 (27.3, 47.2)	17.9 (15.4, 30.9)	15.1 (11.9, 39.9)	0.27
Max. Dev. AP [mm]	58.1 (35.0, 76.7)	45.1 (31.4, 48.7)	38.1 (28.1, 48. 7)	0.39

## DISCUSSION

In this study, examinations of hip muscle strength of the residual limb and examinations of static balance were performed in patients with TFA. The goal was to investigate whether these objective measures could differentiate between patients who were classified at different K-levels. As hypothesized, participants classified at higher K-levels performed better on the strength and balance tests than participants assigned to lower K-levels. However, statistical analyses revealed no significant differences in the parameters between the three K-level groups.

While previous studies showed that participants with TFA suffer from a strength deficit of the residual limb, this is the first study that included an isometric muscle strength assessment of the hip muscles on the affected side for potential K-level distinction. Strength differences were particularly visible between the K3 and the K4 group as well as between the K1/2 and the K4 group. For maximum torque, mean group differences ranged from 0.4 Nm/kg to 0.9 Nm/kg for hip flexion, hip abduction, hip extension and hip adduction. Although not statistically significant, the magnitude of these values implies a distinction between K-level groups.

Heitzmann et al. investigated maximum hip torque in a similar measurement setup and the hip torque differences between participants with TFA (K-level 2 to 4, no differentiation) and healthy participants were in the same numerical range (0.5-0.7 Nm/kg) and statistically significant.^11^ Reasons for the lack of statistical significance in our study may include the small number of participants in the K1/2 and K3 groups, the uneven distribution of participants among K-level groups and the individuality of each participant. Beisheim et al. examined lower extremity strength differences between K3 and K4 classified participants with TFA applying the functional 5-Times Sit-to-Stand Test and found a significant difference.^10^ Functional strength tests were shown to have the potential to differentiate between K-levels. However, they could not explain the reasons why participants of the K3 group performed worse than those assigned to K4. Muscle strength tests of isolated muscle groups of the residual limb, as performed in this study, have the advantage to identify specific muscle weaknesses. Knowing the individual strength deficits are especially important for patients with LLA as hip abductor strength is associated with gait deviations^18^ and hip extension strength has been reported as the greatest predictor of performance on the 6-minute walk test.^19^

As lower extremity strength of lower limb prosthesis users is linked to postural control, parameters of static balance were also investigated to find potential outcome measures that may be able to distinguish between K-levels. The statistical analyses, however, did not reveal significant differences in the parameters across the three K-level groups. Concerning the medians of the parameters, participants classified as K1/2 showed greater COP length as well as greater mean and maximum sway deviations in ML and AP directions in the bipedal stance than the participants of the K3 and K4 groups. For all groups greater sway was observed in the AP than in the ML direction, which has been observed in previous studies.^17^ This may be explained by the missing ankle plantar and dorsiflexor muscles on the amputated leg which are relevant for stability control in the AP direction.^20^

In the eyes-closed condition, COP parameters increased and the differences in COP between K-level groups became larger. Group differences of maximum excursions in AP and ML directions were observed up to 14 mm as well as a mean difference in COP length of 240 mm. Increased COP sway due to the absence of visual input is in line with previous studies investigating patients with LLA during quiet standing.^14,17^ The eyes-closed condition has a great effect on patients with LLA as vision is especially relevant to compensate for balance impairments due to missing somatosensory feedback from the prosthetic leg.^21^ Although not proven in this study, using a closed-eye condition in quiet standing might be a sensitive method for distinguishing between different K-levels as balance control mechanisms differ in relation to functional abilities. Measures of static balance have been criticized that they cannot reflect postural demands in daily life.^22^ However, they may be helpful to identify weaknesses in postural control differentiated in AP or ML directions, which can be relevant for patients with LLA. Static balance tests could be performed in addition to dynamic balance tests, which have been shown to be suitable for K-level distinction among participants with TFA.^10^

Several limitations have to be addressed. The major limitation is the small number of participants in each K-level group as well as the heterogeneity between groups, which may be the reason that no significant differences were detected. The groups differed in age, residual limb length and in post-amputation time, which may have affected the results of physical performance tests. Therefore, generalization of the data is not possible and studies with larger and more homogeneous samples need to confirm the presented results. Further, the cause of amputation was not recorded in this study except that patients who experienced LLA due to diabetes mellitus were excluded. The amputation etiology may impact physical performance and should be recorded in future studies. In balance examinations, prosthetic alignment and different types of prosthetic components (socket, prosthetic knee and foot) may also affect performance and should be controlled in future studies. However, this is the first study that included participants with TFA classified as K1/K2 to find objective measures for supporting K-level classification. Future studies should not only focus on differentiating between participants with LLA classified as K3 and K4 but should also include participants classified as K2.

## CONCLUSION

This study was the first one to perform hip strength tests of the residual limb and static balance tests with participants with TFA classified at different K-levels to find parameters that may be suitable to enhance objective K-level classification. Statistical analyses could not reveal any significant group differences but the value of the magnitude of the group differences detected may be relevant to differentiate between K-level groups. The results of the study suggest that residual limb strength and balance parameters may have the potential to serve as objective measures to support K-level classification but this potential needs to be confirmed by future studies with a larger number of participants.

## DECLARATION OF CONFLICTING INTERESTS

The authors declare that they have no competing interests.

## AUTHOR CONTRIBUTION

- **Stefanie John:** Contributed to the study concept and design, participated in data gathering, analyzed, and interpreted data, contributed to the drafting of the manuscript, read, and approved the final manuscript.
- **Katja Orlowski:** Contributed to the study concept and design, participated in data gathering, contributed to the drafting of the manuscript, read, and approved the final manuscript.
- **Kai-Uwe Mrkor:** Participated in data gathering, contributed to the drafting of the manuscript, read, and approved the final manuscript.
- **Jürgen Edelmann-Nusser:** Contributed to the study concept and design, contributed to the drafting of the manuscript, read, and approved the final manuscript.
- **Kerstin Witte:** Contributed to the study concept and design, analyzed, and interpreted data, contributed to the drafting of the manuscript, read and approved the final manuscript.

## SOURCES OF SUPPORT

German Central Innovation Program for small and medium-sized enterprises for the project ‘Multifunctional diagnostic device for patients of lower limb amputations’ (ZF4096303TS6).

## ETHICAL APPROVAL

The study was approved by the local ethics committee of the Otto von Guericke University Magdeburg and carried out in line with the Declaration of Helsinki (no. of vote: 31/18 on March 19, 2018). Signed informed consent was obtained from all participants.
